# The Framework of Quantifying Biomarkers of OCT and OCTA Images in Retinal Diseases

**DOI:** 10.3390/s24165227

**Published:** 2024-08-13

**Authors:** Xiaoli Liu, Haogang Zhu, Hanji Zhang, Shaoyan Xia

**Affiliations:** 1 School of Biological Science and Medical Engineering, Beihang University, Beijing 100191, China; 2 Hangzhou International Innovation Institute, Beihang University, Beijing 100191, China; 3 School of Medical Technology, Tianjin Medical University, Tianjin 300203, China

**Keywords:** optical coherence tomography angiography (OCTA), quantifying biomarker, retinal diseases, local binary patterns (LBP), foveal avascular zone (FAZ), capillary and large vessel

## Abstract

Despite the significant advancements facilitated by previous research in introducing a plethora of retinal biomarkers, there is a lack of research addressing the clinical need for quantifying different biomarkers and prioritizing their importance for guiding clinical decision making in the context of retinal diseases. To address this issue, our study introduces a novel framework for quantifying biomarkers derived from optical coherence tomography (OCT) and optical coherence tomography angiography (OCTA) images in retinal diseases. We extract 452 feature parameters from five feature types, including local binary patterns (LBP) features of OCT and OCTA, capillary and large vessel features, and the foveal avascular zone (FAZ) feature. Leveraging this extensive feature set, we construct a classification model using a statistically relevant *p* value for feature selection to predict retinal diseases. We obtain a high accuracy of 0.912 and F1-score of 0.906 in the task of disease classification using this framework. We find that OCT and OCTA’s LBP features provide a significant contribution of 77.12% to the significance of biomarkers in predicting retinal diseases, suggesting their potential as latent indicators for clinical diagnosis. This study employs a quantitative analysis framework to identify potential biomarkers for retinal diseases in OCT and OCTA images. Our findings suggest that LBP parameters, skewness and kurtosis values of capillary, the maximum, mean, median, and standard deviation of large vessel, as well as the eccentricity, compactness, flatness, and anisotropy index of FAZ, may serve as significant indicators of retinal conditions.

## 1. Introduction

In the field of ophthalmology, OCT and OCTA have emerged as pivotal tools for visualizing and examining the complex structure of the retina [1]. They not only offer real-time high-resolution volumetric scans of biological tissues but also facilitate the precise extraction of biomarkers associated with these retinal diseases, enabling clinicians to deliver timely and accurate diagnoses and treatment plans. Presently, OCT and OCTA technologies aid in detecting and quantitatively assessing changes in retinal layer thickness, such as the retinal nerve fiber layer and inner retinal layers, which are often closely linked to disease progression [2]. By analyzing vascular density, distribution, and abnormalities, OCTA assists in diagnosing and monitoring various retinal vascular diseases, including diabetic retinopathy and macular degeneration [3]. Moreover, OCT and OCTA provide detailed information about the macular region, including macular thickness, abnormal macular structures, and submacular pathologies [4]. This information is critical for diagnosing and treating retinal diseases, as precise retinal biomarker analysis offers clinicians a comprehensive and in-depth assessment.

Recent studies have highlighted the utility of vascular biomarkers in the diagnosis and management of retinal diseases. A cross-sectional study [5] identifies significant differences in quantitative OCT angiography metrics among individuals with diabetes mellitus (DM) and early stages of diabetic retinopathy (DR), highlighting the potential utility of monitoring disease progression in clinical trials. The systematic literature review [6] explores the impact of retinal biomarkers detected via OCT on disease progression and treatment response in neovascular age-related macular degeneration. Using OCTA, the review consolidates established biomarkers [7] for retinal and choroidal vasculature and evaluates methodological approaches and findings across various retinal diseases. For biomarker research in retinal diseases, OCT angiograms are commonly subjected to quantitative analysis, which involves assessing metrics such as vessel area density, vessel skeleton density, vessel diameter index, vessel perimeter index, and vessel complexity index [8,9,10,11,12,13]. These metrics aid in enhancing the detection and assessment of vascular abnormalities. In addition, fractal dimension [14,15,16,17] has emerged as a pivotal biomarker in the assessment of retinal diseases, offering insights into the complexity and geometric alterations of retinal microvasculature. For the assessment of retinal perfusion and disease severity, biomarkers related to the FAZ have emerged as valuable tools. Research spanning FAZ areas [18,19,20,21,22], FAZ contour irregularity (CI) [23,24,25,26,27], and FAZ perimeter [28,29] has highlighted their significance in clinical evaluation. In the texture feature of OCTA, LBP [30] features are applied to classify spectral domain OCT data, effectively distinguishing diabetic macular edema (DME) patients from normal subjects with high sensitivity and specificity. Additionally, a novel hybrid machine learning approach is proposed for classifying age-related macular degeneration (AMD) using rotation invariant uniform LBP descriptors [31] to capture local texture patterns for feature decorrelation, demonstrating robust performance in distinguishing between different AMD conditions. By leveraging image-based analyses, OCTA facilitates a deeper understanding of retinal microvascular architecture, offering novel insights into disease pathogenesis and progression.

However, existing studies have proposed the utilization of large-scale biomarkers for associating retinal diseases, yet there is a notable absence of quantitative analysis concerning these biomarkers, especially for various types of markers. It is imperative to conduct a comprehensive assessment of the significance of these large-scale biomarkers to determine which ones should be prioritized for analysis in clinical retinal diagnostics. This prioritization would provide invaluable guidance for clinicians in optimizing their diagnostic approaches and enhancing the accuracy and efficacy of retinal disease diagnosis and management.

**Figure 1:**
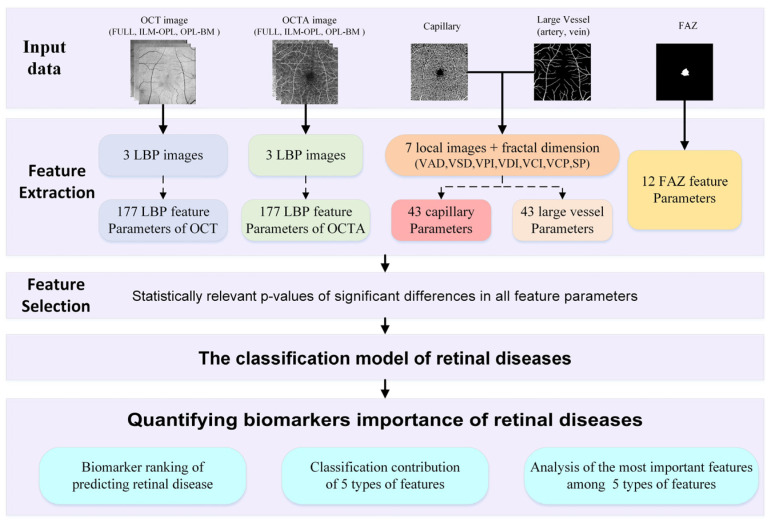
Illustrating the framework of quantified biomarkers in retinal diseases.

To achieve this goal, we propose a framework of quantified biomarkers derived from OCT and OCTA images in retinal diseases, as depicted in Figure 1. Firstly, we extract a comprehensive set of 452 feature parameters from five feature types of aspects: LBP features of OCT and OCTA, capillary and large vessel features, and the FAZ feature, which are closely related to retinal diseases. Leveraging this extensive feature set, we employ a statistically relevant *p* value to select significant differences for feature selection and construct a classification model aimed at predicting retinal diseases. Through this proposed framework of quantified biomarkers, our aim is to classify retinal diseases and rank the biomarkers based on the relative importance of different feature parameters. Additionally, we delve into elucidating the classification contributions and the most important features among the five types of features of retinal diseases.

The main contributions of this paper can be summarized as follows:

(1) We propose a framework for the quantitative analysis of biomarkers associated with retinal diseases. To our knowledge, this is the first study to explore this quantitative analysis framework of retinal biomarkers from five feature types of aspects.
(2) We quantify the significance of biomarkers by machine learning models. This approach systematically analyzes and ranks the importance of biomarkers based on OCT and OCTA images.
(3) We demonstrate that the LBP feature of OCT and OCTA images is among the most crucial biomarkers, potentially serving as latent indicators for the clinical diagnosis of retinal diseases.

## 2. Methods

### 2.1. Data Description

**Figure 2:**
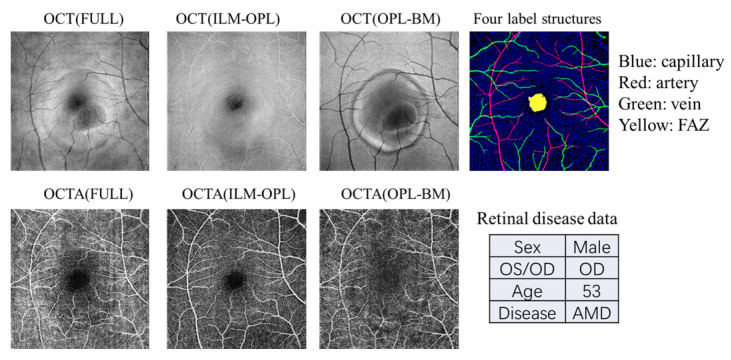
An example of the data types contained in the OCT500 dataset. Each patient includes OCT and OCTA images with three measurement structures: FULL, ILM-OPL, and OPL-BM. Additionally, the dataset provides four binarized structures: capillary, artery, and vein, as well as the FAZ region. The dataset also includes patient demographic information such as gender, age, left or right eye, and disease details.

In this study, we utilize OCT and OCTA data from the OCT500 dataset, derived from a publicly available source [32]. This dataset comprises 500 images containing two modalities of OCT and OCTA with two fields of views (6 mm × 6 mm and 3 × 3 mm). The dataset includes 251 images from normal individuals, 49 images from individuals with AMD, 64 images from individuals with DR, and 136 images from individuals with other retinal diseases. The OCT volumes and OCTA volumes provide both structural and fluid information on the retina, acquired from the same commercial 70 kHz spectral domain OCT system (RTVue-XR, Optovue, Fremont, CA, USA) with a central wavelength of 840 nm. We employ three projection images (FULL, ILM-OPL, and OPL-BM) and four label structures (capillary, artery, vein, and FAZ) associated with various retinal diseases (normal, AMD, DR, and other diseases), as illustrated in the example shown in Figure 2.

### 2.2. Extraction of Feature Parameters Related to Retinal Diseases

In order to explore the quantitative analysis of biomarkers in predicting retinal diseases, we conducted a detailed study by extracting LBP features of OCT and OCTA, vessel features of capillary and large vessel (arterial and vein) features, and FAZ features. The specific feature parameters extracted from these 5 types of features are as follows:

#### 2.2.1. LBP Feature Parameters of OCT and OCTA Images

In OCT images, textures represent variations in reflectivity and backscattering intensity across different retinal layers. These variations can reveal important information about the microstructural organization of the retina, such as the distinct layers of the neurosensory retina, retinal pigment epithelium (RPE), and choroid. Textural patterns observed in OCT images can aid in identifying abnormalities or disruptions in retinal architecture, such as the presence of drusen in AMD or hyperreflective foci in diabetic retinopathy [33]. Similarly, in OCTA images, textures correspond to vascular patterns and flow characteristics within the retinal and choroidal vasculature. OCTA captures dynamic changes in blood flow by detecting motion contrast between consecutive B-scans, allowing for the visualization of retinal vasculature without the need for contrast agents. Texture analysis of OCTA images can help in assessing vascular density, vessel morphology, and perfusion status, providing valuable information for the diagnosis and monitoring of retinal vascular diseases such as retinal vein occlusion and diabetic retinopathy. To utilize these texture features effectively, we extract local binary pattern (LBP) [34] images of OCT and OCTA images to encode the texture information of the retinal region, which is defined as

(1) $$LBP\left(x_{c},y_{c}\right)=\sum_{p=0}^{7}2^{p}\cdot s\left(i_{p}-i_{c}\right),$$

where (*x*_c_,*y*_c_) is central pixel with intensity *i_c_* its neighboring pixel with intensity *i_p_*. *s*(*x*) is the sign function defined as

(2) $$s\left(x\right)=\left\{ \begin{array}{l} 1,x>0\\ 0,others \end{array}\right..$$

**Figure 3:**
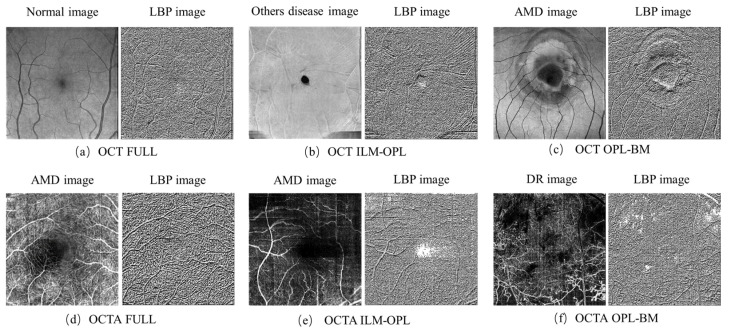
LBP images of FULL, ILM-OPL, and OPL-BM structure in OCT and OCTA images. Normal expression in healthy individuals without retinal diseases, AMD expression in patients with macular degeneration. DR expression in patients with diabetic retinopathy.

**Figure 4:**
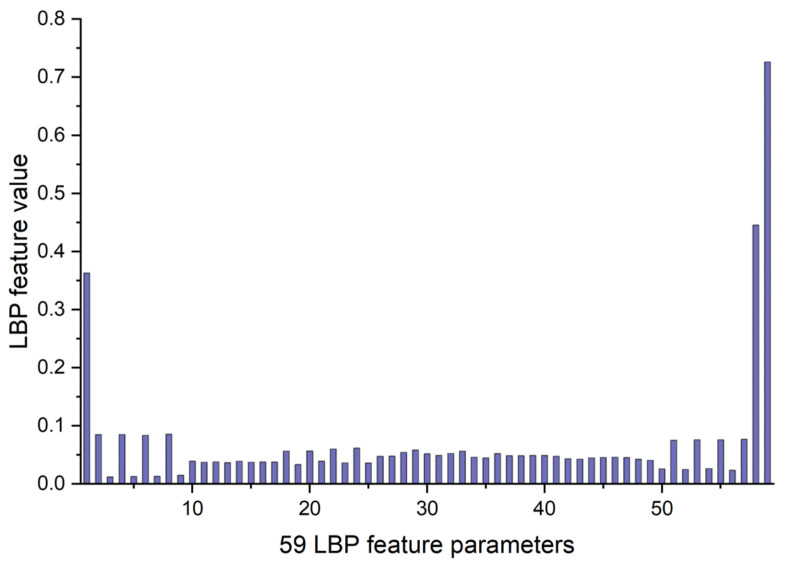
The example of 59 LBP feature parameters for LBP image of OCT FULL from Figure 3a.

The LBP image analysis was successfully performed on the full, ILM-OPL, and OPL-BM structures of OCT and OCTA images for retinal patients using the pipeline outlined in Figure 3. The results demonstrate the effectiveness of LBP analysis in delineating intricate vascular patterns, background texture, and microstructures within retinal images obtained through OCT scans across the three structures: FULL, ILM-OPL, and OPL-BM. Notably, even in instances of low contrast, such as in the OCT ILM-OPL image depicted in Figure 3b, LBP analysis captures local image details. The application of LBP extraction to OCTA images, as depicted in Figure 3d–f, proves particularly valuable in accentuating the rich capillary structure (Figure 3d), delineating the FAZ region (Figure 3e), and highlighting regional structural and textural changes associated with diseases like DR (Figure 3f). LBP analysis plays a critical role in retinal imaging by enhancing the detection and monitoring of subtle textural changes associated with retinal diseases. The method involves comparing each pixel in an image to its surrounding neighborhood and encoding these local structures into a binary code. This process captures variations in retinal reflectivity and vascular perfusion, making it particularly useful for diagnosing conditions like diabetic retinopathy or age-related macular degeneration. By integrating LBP with OCT and OCTA, it becomes possible to observe intricate texture patterns that are often not visible through standard imaging techniques. The LBP-generated histograms, divided into 59 bins corresponding to various texture features (denoted as LBP1, LBP2, …, LBP59), serve as a robust feature vector. This vector effectively encapsulates the texture information across different retinal layers, providing a comprehensive tool for assessing disease progression or treatment response, as depicted in research illustrations like Figure 4.

#### 2.2.2. Vessel Feature of Capillary and Large Vessel

**Figure 5:**
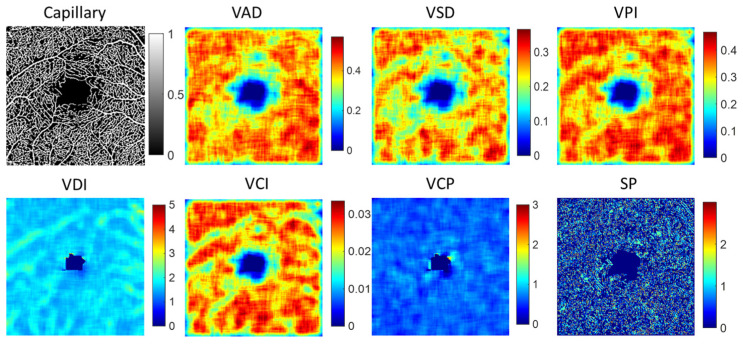
The example of local indices for capillary image. VAD is vessel area density, VSD is vessel skeleton density, VPI is vessel perimeter index, VDI is vessel diameter index, VCI is vessel compactness index, VCP is vessel complexity and SP denotes vessel shape parameter by the curvature.

To analyze alterations in the structure of both capillaries and large blood vessels, we utilized a local window size of 20 × 20 pixels for detailed examination. Several key parameters were extracted to quantify these changes, including vessel area density (VAD), vessel skeleton density (VSD), vessel perimeter index (VPI), vessel diameter index (VDI), and vessel complexity index (VCI). Each parameter has been comprehensively defined in Table 1, providing a clear metric for evaluating the intricacies of vessel architecture. Additionally, we computed the fractal dimension (FD) of vessel structure and measured vessel curvature to depict the vessel shape parameter (SP). Furthermore, we proposed a new feature parameter called vessel complexity (VCP), defined as the ratio of vessel branch points to vessel length, to assess the complexity of the vascular network. Table 1 summarizes the detailed description of the vessel feature, and Figure 5 illustrates an example of local indices for a capillary image. To extract quantitative vascular feature parameters, we calculate the maximum (max), mean, median, standard deviation (std), skewness (skew), and kurtosis (kur) from local VAD, VSD, VPI, VDI, VCI, VCP, and SP images. Combining FD features, we extracted a total of 43 vascular feature parameters from either capillaries or large vessel structures.

#### 2.2.3. FAZ Feature

We quantified changes in the retinal FAZ structure by extracting basic parameters such as FAZ area, perimeter, and circularity index (FAZ CI). Additionally, we extracted the diameter of a circle with the same area as the FAZ region and the centroid parameter representing the geometric center of the FAZ. To quantify the shape of the FAZ region, we designed 6 feature parameters: eccentricity of ellipses with the same second-order moment as the FAZ region, FAZ compactness to quantify the foveal avascular zone’s compactness, FAZ flatness describing the shape of FAZ, FAZ anisotropy index indicating the irregularity of the FAZ region, FAZ convexity representing the proportion of pixels within the region of the convex hull, and FAZ angle describing the directional characteristics of the FAZ region. Table 2 summarizes the detailed description of the 12 feature parameters of FAZ.

### 2.3. Statistical Analyses of Feature Parameters

Statistical analysis was conducted to explore all feature parameters that exhibit significant differences across various groups and may be linked to retinal disease status. Quantitative features for each group underwent a Shapiro–Wilk test to assess normality [35]. Normally distributed variables underwent one-way ANOVA for multiple group comparison, while Student’s *t*-test was utilized for pairwise comparison between control and retinal disease groups. Non-normally distributed features were assessed using the Kruskal–Wallis test for four groups (controls, AMD, DR, and other retinal diseases) and the Mann–Whitney U test for pairwise comparison. Significance for all comparisons was determined at a threshold of *p* < 0.05.

### 2.4. Classification of Machine Learning Models

We conducted the retinal disease classification task using fivefold cross-validation and extracted all feature parameters by eight classification algorithms employed: random forest (RF) [36], extreme gradient boosting decision trees (XGBoost) [37], categorical boosting (Catboost) [38], light gradient boosting machine (LightGBM) [39], support vector machine (SVM) [40], extremely randomized trees (ExtraTrees) [41], Embed Net [42], and Neural Net [43]. The classification process addressed two distinct tasks. Initially, we performed a binary classification task (referred to as 2-class), distinguishing controls from individuals with retinal diseases (1 vs. 1). Subsequently, in the second classification task, a multiclass classification was executed to identify controls, AMD, DR, and other retinal diseases (referred to as 4-class). For each classification task, we partitioned the data into a training set comprising 75% of the data and a testing set comprising the remaining 25%. Feature selection was carried out independently through statistical analyses of feature parameters, using a threshold of *p* < 0.05.

During the nested-cross-validation phase, we employed grid search [44] to identify the most suitable hyperparameters for each model. Within this process, the training set was divided into validation and inner training folds. Subsequently, through inner loops, the validation fold was iteratively shifted along the time dimension. In each iteration, a grid search was conducted, allowing models to be trained. This involved exploring all possible combinations of hyperparameters. Following the training phase, features with importance values below 0.001 were filtered out based on their ranking. This threshold was chosen empirically to ensure that only the most relevant features were retained, while minimizing the inclusion of less informative features. The value of 0.001 was found to provide a good balance between retaining important features and eliminating noise. The models were then retrained using the updated feature set. Those models lacking updated features were considered optimal models. Finally, the optimized model design was trained and tested to determine the final selection.

An Alienware system equipped with a 13th Gen Intel(R) Core (TM) i7-13620H processor running at 2.40 GHz and 32 GB of RAM memory was utilized to execute the overall feature data processing tasks. The initial importing code for converting acquired data was scripted in MATLAB 2023a (MathWorks, Natick, MA, USA), whereas the preprocessing and model classification stages were implemented using Python 3.11.

### 2.5. Performance Evaluation of Retinal Disease Classification

In the classification of retinal diseases utilizing feature parameters extracted from OCT and OCTA images, performance evaluation is crucial. Metrics such as accuracy, precision, sensitivity, and F1-score [45] are commonly employed for this purpose, as defined

(3) $$\begin{array}{l} Accuracy=\frac{TP+TN}{TP+TN+FP+FN},Precision=\frac{TP}{TP+FP}\\ Sensitivity=\frac{TP}{TP+FN},F1-score=\frac{2Precision\times Sensitivity}{Precision+Sensitivity} \end{array}$$

True positive (TP) refers to the number of diseased cases correctly identified as diseased, while true negative (TN) represents the number of healthy cases correctly identified as healthy. False positive (FP) indicates healthy cases incorrectly classified as diseased, and false negative (FN) denotes diseased cases incorrectly classified as healthy. Accuracy (Acc) represents the ratio of correctly classified instances to the total number of instances, providing an overall measure of the model’s correctness. Precision measures the proportion of correctly predicted positive cases out of all cases predicted as positive, indicating the model’s ability to avoid false positives. Sensitivity, also known as recall, measures the proportion of actual positive cases that were correctly identified by the model, reflecting its ability to detect true positives. F1-score, the harmonic means of precision and recall, balances between precision and sensitivity, offering a single metric that considers both false positives and false negatives.

## 3. Results

In this section, we show the results obtained from the process of Figure 1, including the following aspects: (i) LBP feature parameters of OCT and OCTA images; (ii) feature parameters of capillary and large vessel; (iii) FAZ feature parameters; (iv) classification performance of different features for retinal diseases; and (v) quantifying the importance of biomarkers in retinal diseases.

### 3.1. LBP Feature Parameters of OCT and OCTA Images

To efficiently extract LBP feature parameters from LBP images associated with retinal diseases, we obtain 59 LBP parameters, denoted as LBP1, LBP2, …, LBP59, for each LBP image. In this study, LBP feature parameters are extracted from both OCT and OCTA images, focusing on three distinct retinal structures: FULL, ILM-OPL, and OPL-BM. Consequently, each OCT or OCTA image yields a total of 177 LBP parameters (59 LBP parameters per structure, multiplied by 3 structures). For further data details, LBP feature data for OCT and OCTA are provided in Supplementary Data S1. To select valuable LBP parameters for predicting retinal diseases, Table 3 presents the statistical *p* value of the 10 most significant differences with *p* value < 0.05 in LBP feature parameters (the complete LBP feature data significant differences are presented in Supplementary Data S2). From the displayed values, it can be seen that regardless of OCT or OCTA images, the ILM-OPL and OPL-BM structural images provide a substantial number of LBP feature parameters with significant differences in control groups and retinal diseases.

### 3.2. Feature Parameters of Capillary and Large Vessel

To investigate the significance of vessel features in retinal diseases, each vessel structure yields 43 vessel feature parameters (7 vessel features per index, multiplied by 6 indices, plus vessel FD). This comprehensive analysis enables a thorough investigation into the role of vessel features in retinal diseases. Table 4 shows the statistical *p* value of the 10 most significant differences with *p* value < 0.05 in capillary and large vessel structures (the complete vessel feature data significant differences are presented in Supplementary Data S2). From the numerical values, it is evident that the most notable vessel feature parameters are concentrated within the local indices of VSD, VAD, VCI, and VPI. However, capillaries exhibit significant features primarily in skewness, kurtosis, and median values, whereas large vessels demonstrate significant features mainly in standard deviation, maximum, mean, and median values.

### 3.3. FAZ Feature Parameters

To extract quantitative features of FAZ, we calculate 12 feature parameters of FAZ, including FAZ region, FAZ perimeter, diameter, FAZ centroid coordinates *x* and *y*, eccentricity, etc. The specific values of all FAZ parameters are shown in Supplementary Data S3. The statistical significance of differences with *p* value < 0.05 for FAZ features is outlined in Table 5. It is noteworthy that besides the commonly utilized FAZ area, perimeter, and FAZ-CI, several other parameters also hold significance in evaluating retinal diseases, including FAZ anisotropy index, flatness, eccentricity, convexity, compactness, diameter, and centroid coordinate y. Together, these metrics provide a holistic perspective on FAZ, thereby enhancing our comprehension of retinal pathologies and enabling more accurate diagnosis and treatment strategies.

### 3.4. Classification Performance of Different Features for Retinal Diseases

Based on the above results, we extracted 452 feature parameters from 5 aspects, including: 177 LBP parameters of OCT images, 177 LBP parameters of OCTA, 43 capillary parameters, 43 large vessel parameters, and 12 FAZ parameters. To analyze the specific value of these feature parameters in retinal diseases, we first implemented feature selection based on *p* values < 0.05, indicating significant differences as observed in the results of Section 3.1, Section 3.2 and Section 3.3. Subsequently, we performed two distinct classification tasks using eight machine learning classification algorithms: RF, XGBoost, Catboost, LightGBM, SVM, ExtraTrees, Embed Net, and Neural Net. Initially, we performed a binary classification task (denoted as 2-class), distinguishing between controls and individuals with retinal diseases (1 vs. 1). Subsequently, in the second classification task, we conducted a multiclass classification where the classifier identified controls, AMD, DR, and other retinal diseases (denoted as 4-class). The classification results, reported in Table 6, detail the performance of eight different models across various features for retinal disease classification.

In terms of classification accuracy across both 2-class and 4-class tasks, most models ranked the five feature categories as follows: LBP of OCT > LBP of OCTA > capillary > large vessel > FAZ. Moreover, the result demonstrates higher accuracy when utilizing LBP features compared to those of capillary, large vessel, and FAZ. These findings indicate that OCT and OCTA contain numerous potential markers crucial for predicting retinal diseases. Notably, the classification performance reaches its peak when all features are used. Specifically, in the binary classification task (distinguishing between control and diseased groups), the RF model achieved the highest performance with an accuracy of 0.912, a sensitivity of 0.855, and an F1-score of 0.906. Similarly, in the multiclass classification task (identifying controls, AMD, DR, and other retinal diseases), the RF model again showed the highest performance with an accuracy of 0.752, a precision of 0.769, and a sensitivity of 0.752. This indicates that combining all five types of features significantly enhances the classification performance of retinal diseases.

### 3.5. Quantifying the Importance of Biomarkers in Retinal Diseases

#### 3.5.1. Biomarker Ranking of Predicting Retinal Diseases

**Figure 6:**
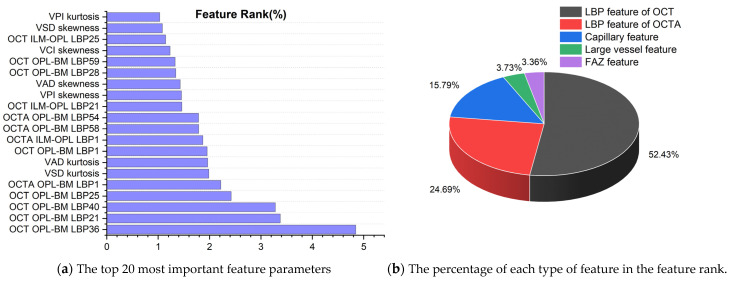
The feature ranking results of random forest model in binary classification of retinal diseases.

To analyze the contribution of different features to the classification of retinal diseases, we selected the RF model with the highest classification performance in the retinal binary classification task for quantitative analysis, and the results are shown in Figure 6a. From the top 20 most important parameters (all feature ranking data used for retinal prediction are presented in Supplementary Data S4), it can be seen that most of them are LBP feature parameters of OPL-BM and ILM-OPL structural images for OCT and OCTA, indicating that LBP of OPL-BM and ILM-OPL plays the most important role in predicting retinal diseases. In addition, the kurtosis and skewness values of VAD, VSD, and VPI also occupy a significant proportion of the feature rankings, emphasizing their importance in capillary analysis for accurate retinal disease classification.

#### 3.5.2. Classification Contribution of 5 Types of Features

To explore the significance of capillary, large vessel, FAZ, LBP of OCT, and OCTA features in predicting retinal diseases, Figure 6b presents the percentage distribution of different features in the ranking obtained from the RF model. The graph reveals that the LBP feature of OCT holds the highest proportion in predicting retinal diseases, accounting for 52.43%, followed by the LBP feature of OCTA at 24.69%. Additionally, the capillary feature contributes 15.79%, while the large vessel feature occupies 3.73% of the ranking. The FAZ feature exhibits the lowest proportion at 3.36%. Notably, LBP features extracted from OCT and OCTA images exhibit significantly higher importance in the ranking compared to other feature types. This underscores the pivotal role of LBP features, suggesting their superior informativeness and discriminative power. These findings highlight the abundance of LBP features in OCT and OCTA images, providing valuable insights for predicting retinal diseases.

#### 3.5.3. Analysis of the Most Important Features among 5 Types of Features

What are the respective parameters of capillary, large vessel, FAZ, LBP of OCT and OCTA structures that have the ability to predict retinal diseases? To answer this question, Table 7 presents the top 10 feature parameter importance values for each type of feature (the complete data are presented in Supplementary Data S5).

In the realm of OCT features, LBP descriptors stand out significantly, particularly in the OPL-BM structural image. Key parameters such as LBP36, LBP21, LBP40, LBP25, and LBP1 emerge as pivotal predictors. Turning to OCTA features, OPL-BM LBP1, ILM-OPL LBP1, OPL-BM LBP58, and OPL-BM LBP54 are prominently ranked for their importance. LBP derived from the OPL-BM and ILM-OPL structural images in OCT and OCT angiography (OCTA) provides valuable features for analyzing retinal diseases. These layers—OPL (outer plexiform layer) to BM (Bruch’s membrane) and ILM (inner limiting membrane) to OPL—represent key segments of the retina where disease-related changes can be pronounced. The LBP features extracted from these segments effectively capture textural alterations that are critical for diagnosing and monitoring the progression of retinal pathologies. By focusing on these specific layers, the LBP analysis helps highlight subtle yet significant variations in retinal structure, which are essential for early detection and treatment strategies.

Capillary parameters reveal notable significance, with the kurtosis values of VSD and VAD, as well as the skewness values of VPI, VAD, and VCI, taking the spotlight. In the domain of large vessel features, critical parameters include the average value of VSD, the standard deviation and maximum value of VCI, alongside the average value of VAD, and the standard deviation and median value of VPI. From the contrast result, it is clear that the feature parameters VSD, VAD, VPI, and VCI are critical in analyzing both capillary and large vessel characteristics, emphasizing the importance of vessel density, skeleton density, perimeter, and vessel complexity in describing retinal diseases. These parameters offer valuable insights into the vascular aspects of the retina, which are essential for understanding and diagnosing retinal conditions. Furthermore, the specific metrics for capillaries and large vessels differ, which underscores the heterogeneity in their structural and functional roles within the retinal vascular network. Capillaries tend to be characterized more by kurtosis and skewness, which describe the tailness and asymmetry of the distribution of their respective features. In contrast, large vessels are primarily analyzed through mean, standard deviation (std), and maximum (max) values, highlighting differences in average diameter, variability, and peak size, respectively. This differentiation between capillary and large vessel features not only enriches the understanding of retinal vascular anatomy but also implies that a more detailed segmentation into capillary and large vessel categories could significantly enhance the prediction and diagnosis of retinal diseases. By targeting specific vessel types, it may be possible to develop more precise and effective diagnostic tools tailored to the unique pathophysiological changes associated with different retinal vascular components.

FAZ features provide valuable insights in retinal binary classification, particularly in eccentricity, FAZ compactness, FAZ flatness, and FAZ anisotropy index. Moreover, these specific FAZ features offer a more refined analysis compared to commonly used metrics like FAZ area and perimeter. While area and perimeter give a basic understanding of the size and outline of the FAZ, additional parameters like eccentricity, compactness, and flatness. This detailed analysis makes them more indicative of subtle FAZ pathological changes, enhancing the diagnostic precision for retinal diseases.

The analysis presented in this study clearly demonstrates that the retinal biomarker framework we designed has successfully identified numerous previously unrecognized retinal feature parameters. These parameters hold promise as potential biomarkers for the diagnosis of retinal diseases, representing a significant advancement over traditional diagnostic methods. By providing a deeper understanding of the pathological mechanisms involved, this innovation not only enhances our capability to detect retinal pathologies but also facilitates improved patient outcomes through more timely and precise retinal diagnoses.

## 4. Discussion

This study establishes a framework of feature biomarkers for the prediction of retinal diseases, encompassing five types of features: LBP feature extracted from OCT and OCTA images, vessel feature derived from capillary and large vessel, and FAZ feature. This comprehensive analysis aims to enhance the accuracy of retinal disease prediction.

Through thorough comparison, our findings indicate that capillaries offer superior classification performance compared to large vessels in the task of retinal disease classification. This observation may be attributed to the fact that many retinal diseases, such as diabetic retinopathy and retinal vein occlusion [46], often manifest initially at the capillary level. Capillaries, with their thinner walls, are more susceptible to inflammation, hypoxia, and other forms of injury, thus predisposing them to microvascular lesions [47]. Consequently, alterations in the morphology and function of capillaries may serve as early indicators of disease development and severity [48], outperforming changes observed in vessels.

Furthermore, our investigation revealed that the utilization of OCT and OCTA imaging, which yield a plethora of LBP feature parameters, resulted in the highest classification performance for classification tasks involving retinal diseases. This notable outcome may be attributed to the comprehensive nature of OCT and OCTA images, which capture not only diverse vascular structures but also intricate texture patterns, providing a rich representation of retinal pathology. By leveraging the complex texture structures present in OCT and OCTA images, coupled with the discriminative power of LBP features, we can achieve superior classification performance in the prediction and management of retinal conditions.

This study quantifies their contributions as feature biomarkers in predicting retinal diseases through a comprehensive analysis of LBP features of OCT and OCTA images, capillary and large vessel characteristics, and FAZ. Notably, the binary classification of retinal diseases highlights the significance of specific features within OCT OPL-BM images. LBP36, LBP21, LBP40, and LBP25 from OCT OPL-BM images, alongside LBP1 from OCTA OPL-BM images, emerge as the top 5 most influential features. These findings, presented in Figure 6a, underscore their discernible capacity in distinguishing between control subjects and those with retinal diseases. Thus, these identified top 5 features hold promise as potential biomarkers for diagnosing retinal disease. Furthermore, it is noteworthy that within the characteristics of capillaries, the skewness and kurtosis of VSD, VAD, VPI, and VCI exhibit greater importance than mean and max values. This emphasis on skewness and kurtosis, which depict the distribution of data, suggests that the distribution characteristics of VSD, VAD, VPI, and VCI in capillaries hold significant diagnostic value for retinal diseases. In contrast, the features of large vessels predominantly center around the max, mean, median, and standard deviation of VCI, VAD, and VPI. This indicates that for large vessels, the focus is more on the local structural properties rather than the overall distribution. Comparing these vascular markers clearly shows significant differences between capillary and large vessel markers in predicting retinal diseases, highlighting that capillary analysis is more concerned with the entire distribution structure, whereas large vessel analysis focuses more on specific local attributes. Therefore, the specific differentiation of vascular characteristics into capillaries and large vessels in this paper is beneficial for predicting retinal diseases.

Regarding FAZ features, it is observed that FAZ area and FAZ CI are not the primary predictors; instead, parameters such as eccentricity, FAZ compactness, FAZ flatness, and FAZ anisotropy index demonstrate substantial predictive value. These metrics offer nuanced perspectives on the shape of the FAZ region within the retina. Eccentricity, a measure of how much the shape of the FAZ deviates from a perfect circle, provides crucial information on the irregularities present in the central avascular zone. In the context of retinal diseases, such as diabetic retinopathy or macular degeneration, alterations in the FAZ’s contour often signify pathological changes, ranging from mild distortions to pronounced elongations or contractions [49]. Compactness, quantifying the density of the FAZ area relative to its boundary length, delves into the intricacies of how tightly or loosely the FAZ region is packed. In diseased retinas, disruptions to the FAZ boundary can result in irregular compactness values [50], reflecting changes in the spatial arrangement and density of retinal structures within the FAZ. FAZ flatness, elucidating the degree of planarity or curvature within the FAZ area, unveils insights into the morphology of the central avascular zone [51]. Anomalous flatness values may indicate abnormalities in retinal architecture, such as thinning or thickening of the FAZ region, which are characteristic features of various retinal diseases. The FAZ anisotropy index, gauging the degree of structural orientation or alignment within the FAZ region, uncovers subtle variations in tissue organization and microarchitecture. Perturbations in the FAZ’s internal structure [52], attributable to conditions like retinal vascular disorders or ischemic retinopathies, can manifest as alterations in anisotropy indices, reflecting changes in the directional coherence of retinal tissues. The prominence of these FAZ metrics in predicting retinal diseases underscores their potential as sensitive indicators of pathological changes within the retina. By capturing the nuanced alterations in FAZ morphology and microstructure, these metrics offer valuable insights into disease progression and may pave the way for more accurate diagnostic and prognostic assessments in clinical practice.

Future studies should consider incorporating alternative feature selection methods, such as LASSO (least absolute shrinkage and selection operator) and RFE (recursive feature elimination), as well as model optimization techniques including hyperparameter tuning. These approaches can potentially enhance the performance and robustness of the models. While the proposed framework demonstrates promising results, further validation in real clinical settings is necessary to confirm its effectiveness and reliability in practical applications. Future studies should prioritize clinical validation to ensure the method’s robustness and applicability in real-world scenarios.

## 5. Conclusions

In the present work, we conducted a comprehensive analysis of five distinct types of features to predict retinal diseases, leveraging biomarkers derived from OCT and OCTA images. Our research presents a novel framework for the quantitative analysis of these biomarkers, marking the first study to explore such a framework from multiple feature aspects. Within this framework, we systematically quantified and ranked the importance of these biomarkers, providing a robust method for their evaluation.

One of the key findings of our work is the identification of the LBP feature from OCT and OCTA images as a critical biomarker. This discovery highlights the potential of LBP features as latent indicators for the clinical diagnosis of retinal diseases. Additionally, we found that the skewness and kurtosis of VSD, VAD, VPI, and VCI suggest that the global distribution characteristics of capillaries are crucial for diagnosis. For large vessels, the mean, max, median, and standard deviation of VCI, VAD, and VPI emphasize local vessel structural properties. Furthermore, our analysis revealed that FAZ provides valuable descriptive parameters such as eccentricity, FAZ compactness, FAZ flatness, and FAZ anisotropy index, which are significant for characterizing FAZ region conditions.

Our quantitative analysis not only underscores the significance of these biomarkers but also identifies the most crucial predictors among them, offering a detailed hierarchy of their importance. This comparison highlights significant differences between LBP of OCT and OCTA, capillary, large vessel, and FAZ.

This multifaceted examination not only enhances our understanding of retinal biomarkers but also offers valuable insights that could inform further research and clinical applications. By advancing the methodologies for analyzing retinal biomarkers, our study paves the way for more accurate and effective diagnosis and management of retinal diseases, ultimately contributing to better patient outcomes and advancing the field of ophthalmology.

## Figures and Tables

**Table 1:** Summary of vessel features on capillary and large vessel.

Vessel Features	Definition	Description
Fractal dimension (FD)	$-\log N_{r}/\log r$	Measured the complexity or irregularity of vessel structure.
Vessel area density (VAD)	The ratio of the vessel area to local window	Quantified the proportion of the area occupied by blood vessels, providing a measure of vascular density.
Vessel skeleton density (VSD)	The ratio of the total length of the vessel skeleton to local window	Quantified the density of the vessel network, providing insights into the vascular branching and connectivity.
Vessel perimeter index (VPI)	The ratio of vessel perimeter to local window	Quantified the length of the vessel boundary, providing insights into the morphology and branching patterns.
Vessel diameter index (VDI)	VAD/VSD	Quantified the relationship between the density of blood vessels and the density of the vessel skeleton.
Vessel complexity index (VCI)	$VPI^{2}/\left(4\pi \times VAD\right)$	Quantifies the complexity of blood vessels by assessing the relationship between vessel perimeter, vessel area density, and the circularity of vessels.
Vessel complexity (VCP)	The ratio of the number of branch points to the vessel length	Measured of the complexity of the vascular network.
Shape (SP)	$\frac{x^{\prime }\left(s\right)y^{\prime \prime }\left(s\right)-y^{\prime }\left(s\right)x^{\prime \prime }\left(s\right)}{{\left[{x^{\prime }}^{2}\left(s\right)+{y^{\prime }}^{2}\left(s\right)\right]}^{3/2}}$	Quantified the degree of bending or deviation from a straight path along their length.

**Table 2:** Summary of 12 feature parameters on FAZ region.

FAZ Features	Definition	Description
FAZ area	The sum of pixels in the FAZ region	The avascular region at the center of the fovea in the retina.
FAZ perimeter	Distance around the boundary of the FAZ region	Measurements of FAZ size
FAZ CI	$4\pi \times area_{FAZ}/perimeter_{FAZ}^{2}$	Measured the circularity of the FAZ, with values nearing 1 indicating a circular shape and deviations from 1 indicating irregularities.
Diameter	$2\sqrt{area_{FAZ}/\pi }$	The diameter of a circle with the same area as the FAZ region
FAZ centroid coordinatesx and y	The centroid of the FAZ region	The centroid parameter of the FAZ region represents the geometric center of the foveal avascular zone
Eccentricity	Eccentricity of ellipses with the same second-order moment as the FAZ region	Measured the degree of deviation from a perfect circle in the shape of the foveal avascular zone.
FAZ compactness	The ratio of the number of pixels in the FAZ region to the total number of pixels in the bounding box	Quantified the compactness of the foveal avascular zone. A value closer to 1 indicates a more compact FAZ region with a concentrated pixel distribution, while a smaller value suggests a more dispersed area.
FAZ flatness	The approximate ellipse’s minor axis divided by the major axis in the FAZ region	Described shape of FAZ, allowing evaluation of whether the FAZ region exhibits an elliptical shape.
FAZ anisotropy index	$Perimeter_{FAZ}/\;\left(\pi \times Diameter\right)$	This ratio measures the deviation of the foveal avascular zone’s perimeter from that of an ideal circle, indicating the irregularity of the FAZ region’s shape
FAZ Convexity	Area/convex area	The proportion of pixels within the FAZ region of the convex hull.
FAZ angle	The inclination angle of FAZ approximate ellipse	Described the directional characteristic of the FAZ region

**Table 3:** Statistically relevant *p* value of 10 most significant differences in LBP feature parameters for OCT and OCTA image with three distinct retinal structures: FULL, ILM-OPL, and OPL-BM.

OCT LBP Parameters	Control	Retinal Disease	*p* Value	OCTA LBP Parameters	Control	Retinal Disease	*p* Value
OPL-BM LBP36	0.046	0.058	2.95 × 10^−52^	OPL-BM LBP1	0.368	0.345	1.25 × 10^−42^
OPL-BM LBP21	0.037	0.045	2.48 × 10^−48^	OPL-BM LBP58	0.464	0.503	2.97 × 10^−41^
OPL-BM LBP40	0.045	0.055	1.95 × 10^−47^	OPL-BM LBP50	0.022	0.025	1.14 × 10^−39^
OPL-BM LBP25	0.036	0.043	2.27 × 10^−41^	OPL-BM LBP54	0.022	0.025	2.37 × 10^−39^
ILM-OPL LBP21	0.034	0.041	1.96 × 10^−39^	OPL-BM LBP52	0.021	0.025	3.74 × 10^−39^
OPL-BM LBP29	0.055	0.068	1.08 × 10^−36^	OPL-BM LBP56	0.022	0.025	4.32 × 10^−37^
ILM-OPL LBP25	0.032	0.038	5.51 × 10^−35^	ILM-OPL LBP1	0.369	0.346	1.87 × 10^−35^
ILM-OPL LBP29	0.048	0.058	1.29 × 10^−32^	OPL-BM LBP55	0.080	0.074	1.26 × 10^−33^
OPL-BM LBP28	0.055	0.067	3.26 × 10^−32^	ILM-OPL LBP58	0.463	0.501	4.53 × 10^−33^
OPL-BM LBP33	0.054	0.065	8.59 × 10^−32^	OPL-BM LBP7	0.017	0.015	1.93 × 10^−31^

**Table 4:** Statistically relevant *p* value of 10 most significant differences in vessel feature parameters for capillary and large vessel.

Capillary Feature	Control	Retinal Disease	*p* Value	Large Vessel Feature	Control	Retinal Disease	*p* Value
VSD skewness	−1.157	−0.514	3.74 × 10^−36^	VCI std	0.0010	0.0012	9.11 × 10^−22^
VAD skewness	−1.575	−0.804	4.37 × 10^−36^	VSD std	0.0099	0.0117	1.34 × 10^−20^
VCI skewness	−1.114	−0.504	4.98 × 10^−35^	VPI std	0.0180	0.0208	7.07 × 10^−20^
VPI skewness	−1.541	−0.813	6.04 × 10^−35^	VCI max	0.0054	0.0063	3.25 × 10^−17^
VAD kurtosis	6.325	4.027	6.46 × 10^−32^	VSD max	0.0543	0.0625	5.99 × 10^−17^
VPI kurtosis	6.082	3.829	2.12 × 10^−31^	VPI max	0.0972	0.1100	6.49 × 10^−16^
VSD kurtosis	4.898	3.311	5.72 × 10^−31^	VCI mean	0.0025	0.0029	3.33 × 10^−15^
VCI kurtosis	4.481	3.077	4.16 × 10^−27^	VPI median	0.0446	0.0526	3.92 × 10^−15^
VSD median	0.256	0.210	2.81 × 10^−26^	VCI median	0.0025	0.0030	4.26 × 10^−15^
VPI median	0.360	0.303	5.85 × 10^−26^	VSD median	0.0248	0.0294	4.45 × 10^−15^

**Table 5:** Statistically relevant *p* value of significant differences in all FAZ feature parameters.

FAZ Feature	Control	Retinal Disease	*p* Value
FAZ CI	0.607	0.514	4.63 × 10^−14^
FAZ anisotropy index	1.306	1.446	1.42 × 10^−12^
FAZ area	0.027	0.018	7.52 × 10^−10^
FAZ flatness	0.874	0.817	9.41 × 10^−10^
Eccentricity	0.465	0.547	9.41 × 10^−10^
FAZ Convexity	0.870	0.845	2.26 × 10^−07^
FAZ compactness	0.633	0.607	2.6 × 10^−06^
Diameter	57.096	52.078	6.56 × 10^−05^
FAZ perimeter	0.721	0.646	9.98 × 10^−05^
FAZ centroid coordinates y	0.498	0.507	0.004773

**Table 6:** Classification results of 5 types of features (capillary, large vessel, FAZ, LBP of OCT, and OCTA) with two classification tasks.

Classification Model	Different Features	2-Class (Control and Retinal Disease)				4-Class (Control, AMD, DR and Others)			
		Accuracy	Precision	Sensitivity	F1-Score	Accuracy	Precision	Sensitivity	F1-Score
Random Forest	All features	0.912	0.964	0.855	0.906	0.752	0.769	0.752	0.716
	Capillary	0.800	0.768	0.855	0.809	0.592	0.601	0.592	0.590
	Large vessel	0.776	0.718	0.903	0.800	0.568	0.578	0.568	0.546
	FAZ	0.744	0.703	0.839	0.765	0.584	0.597	0.584	0.572
	LBP feature of OCT	0.824	0.900	0.726	0.804	0.688	0.672	0.688	0.668
	LBP feature of OCTA	0.792	0.773	0.823	0.797	0.608	0.623	0.608	0.591
XGBoost	All features	0.904	0.981	0.823	0.895	0.728	0.729	0.728	0.712
	Capillary	0.744	0.727	0.774	0.750	0.600	0.593	0.600	0.595
	Large vessel	0.720	0.685	0.806	0.741	0.496	0.498	0.496	0.495
	FAZ	0.704	0.676	0.774	0.722	0.560	0.527	0.560	0.536
	LBP feature of OCT	0.840	0.904	0.758	0.825	0.656	0.634	0.656	0.640
	LBP feature of OCTA	0.752	0.718	0.823	0.767	0.608	0.562	0.608	0.581
Catboost	All features	0.896	0.980	0.806	0.885	0.720	0.714	0.720	0.706
	Capillary	0.760	0.735	0.806	0.769	0.592	0.591	0.592	0.588
	Large vessel	0.760	0.711	0.871	0.783	0.520	0.537	0.520	0.510
	FAZ	0.728	0.712	0.758	0.734	0.536	0.512	0.536	0.520
	LBP feature of OCT	0.848	0.978	0.710	0.822	0.680	0.669	0.680	0.672
	LBP feature of OCTA	0.816	0.800	0.839	0.819	0.672	0.651	0.672	0.654
LightGBM	All features	0.896	0.980	0.806	0.885	0.728	0.725	0.728	0.708
	Capillary	0.792	0.773	0.823	0.797	0.568	0.551	0.568	0.555
	Large vessel	0.704	0.676	0.774	0.722	0.480	0.459	0.480	0.468
	FAZ	0.720	0.701	0.758	0.729	0.592	0.623	0.592	0.580
	LBP feature of OCT	0.824	0.917	0.710	0.800	0.688	0.666	0.688	0.673
	LBP feature of OCTA	0.800	0.768	0.855	0.809	0.608	0.604	0.608	0.594
SVM	All features	0.848	0.906	0.774	0.835	0.648	0.650	0.648	0.648
	Capillary	0.768	0.739	0.823	0.779	0.624	0.662	0.624	0.635
	Large vessel	0.736	0.699	0.823	0.756	0.352	0.475	0.352	0.387
	FAZ	0.752	0.816	0.645	0.721	0.552	0.612	0.552	0.574
	LBP feature of OCT	0.848	0.922	0.758	0.832	0.600	0.616	0.600	0.607
	LBP feature of OCTA	0.848	0.864	0.823	0.843	0.608	0.636	0.608	0.617
ExtraTrees	All features	0.896	0.945	0.839	0.889	0.704	0.741	0.704	0.661
	Capillary	0.752	0.738	0.774	0.756	0.632	0.607	0.632	0.607
	Large vessel	0.752	0.701	0.871	0.777	0.536	0.539	0.536	0.501
	FAZ	0.752	0.731	0.790	0.760	0.536	0.491	0.536	0.507
	LBP feature of OCT	0.840	0.904	0.758	0.825	0.696	0.690	0.696	0.671
	LBP feature of OCTA	0.792	0.757	0.855	0.803	0.600	0.572	0.600	0.569
Embed Net	All features	0.864	0.941	0.774	0.850	0.728	0.751	0.728	0.717
	Capillary	0.744	0.714	0.806	0.758	0.592	0.589	0.592	0.590
	Large vessel	0.760	0.705	0.887	0.786	0.464	0.465	0.464	0.464
	FAZ	0.696	0.688	0.710	0.698	0.472	0.472	0.472	0.472
	LBP feature of OCT	0.832	0.902	0.742	0.814	0.672	0.666	0.672	0.663
	LBP feature of OCTA	0.784	0.787	0.774	0.780	0.624	0.602	0.624	0.609
Neural Net	All features	0.856	1.000	0.710	0.830	0.744	0.755	0.744	0.725
	Capillary	0.768	0.780	0.742	0.760	0.568	0.564	0.568	0.563
	Large vessel	0.688	0.689	0.677	0.683	0.448	0.440	0.448	0.444
	FAZ	0.728	0.684	0.839	0.754	0.544	0.564	0.544	0.552
	LBP feature of OCT	0.816	0.898	0.710	0.793	0.704	0.696	0.704	0.684
	LBP feature of OCTA	0.792	0.800	0.774	0.787	0.616	0.600	0.616	0.605

**Table 7:** The top 10 most important parameters of 5 types of features (capillary, large vessel, FAZ, LBP of OCT and OCTA) in binary classification of retinal disease via random forest (RF) model.

LBP Parameters of OCT	Feature Importance (%)	LBP Parameters of OCTA	Feature Importance (%)	Capillary Feature	Feature Importance (%)
OPL-BM LBP36	4.847	OPL-BM LBP1	2.220	VSD kurtosis	1.986
OPL-BM LBP21	3.377	ILM-OPL-LBP1	1.870	VAD kurtosis	1.964
OPL-BM LBP40	3.282	OPL-BM LBP58	1.789	VPI skewness	1.455
OPL-BM LBP25	2.421	OPL-BM LBP54	1.786	VAD skewness	1.429
OPL-BM LBP1	1.954	OPL-BM LBP52	0.955	VCI skewness	1.232
ILM-OPL LBP21	1.461	ILM-OPL LBP58	0.853	VSD skewness	1.083
OPL-BM LBP28	1.343	OPL-BM LBP50	0.761	VPI kurtosis	1.032
OPL-BM LBP59	1.330	OPL-BM LBP56	0.748	VCI median	0.643
ILM-OPL LBP25	1.146	OPL-BM LBP7	0.615	VSD mean	0.565
OPL-BM LBP33	0.954	OPL-BM LBP35	0.585	VPI mean	0.560
Large Vessel Parameters	Feature Importance (%)	FAZ Parameters	Feature Importance (%)		
VSD mean	0.426	Eccentricity	0.564		
VCI std	0.357	FAZ compactness	0.517		
VCI max	0.299	FAZ flatness	0.479		
VAD mean	0.236	FAZ anisotropy index	0.430		
VPI std	0.222	FAZ perimeter	0.398		
VPI median	0.213	FAZ CI	0.372		
VCP kurtosis	0.202	FAZ centroid coordinates y	0.214		
VSD skewness	0.201	FAZ area	0.195		
VCI kurtosis	0.200	Diameter	0.186		
VCI mean	0.199				

## Data Availability

https://ieee-dataport.org/open-access/octa-500 (accessed on 1 January 2024).

## Supplementary Materials

The following supporting information can be downloaded at: https://www.mdpi.com/article/10.3390/s24165227/s1.

## References

[1] Huang K., Ma X., Zhang Z., Zhang Y., Yuan S., Fu H., Chen Q. (2024). Diverse Data Generation for Retinal Layer Segmentation with Potential Structure Modelling. IEEE Trans. Med. Imaging.

[2] Kashani A.H., Chen C.L., Gahm J.K., Zheng F., Richter G.M., Rosenfeld P.J., Wang R.K. (2017). Optical Coherence Tomography Angiography: A Comprehensive Review of Current Methods and Clinical Applications. Prog. Retin. Eye Res..

[3] Mastropasqua R., Antonio L.D., Staso S.D., Agnifili L., Gregorio A.D., Ciancaglini M., Mastropasqua L. (2015). Optical Coherence Tomography Angiography in Retinal Vascular Diseases and Choroidal Neovascularization. J. Ophthalmol..

[4] Uji A., Balasubramanian S., Lei J., Baghdasaryan E., Al-Sheikh M., Sadda S.R. (2017). Impact of Multiple En Face Image Averaging on Quantitative Assessment from Optical Coherence Tomography Angiography Images. Ophthalmology.

[5] Hogg R.E., Wright D.M., Dolz-Marco R., Gray C., Waheed N., Teussink M.M., Naskas T., Perais J., Das R., Quinn N. (2021). Quantitative Parameters from OCT Angiography in Patients with Diabetic Retinopathy and in Those with Only Peripheral Retinopathy Compared with Control Participants. Ophthalmol. Sci..

[6] Hanson R.L., Airody A., Sivaprasad S., Gale R.P. (2023). Optical Coherence Tomography Imaging Biomarkers Associated with Neovascular Age-Related Macular Degeneration: A Systematic Review. Eye.

[7] Kalra G., Zarranz-Ventura J., Chahal R., Bernal-Morales C., Lupidi M., Chhablani J. (2022). Optical Coherence Tomography (OCT) Angiolytics: A Review of OCT Angiography Quantitative Biomarkers. Surv. Ophthalmol..

[8] Chu Z., Lin J., Gao C., Xin C., Zhang Q., Chen C.L., Roisman L., Gregori G., Rosenfeld P.J., Wang R.K. (2016). Quantitative Assessment of the Retinal Microvasculature Using Optical Coherence Tomography Angiography. J. Biomed. Opt..

[9] Zhao Q., Yang W.L., Wang X.N., Wang R.K., You Q.S., Chu D.Z., Xin C., Zhang M.Y., Li D.J., Wang Z.Y. (2018). Repeatability and Reproducibility of Quantitative Assessment of the Retinal Microvasculature Using Optical Coherence Tomography Angiography Based on Optical Microangiography. Biomed. Environ. Sci..

[10] Yan Y., Zhou X., Chu Z., Stell L., Shariati M.A., Wang R.K., Liao Y.J. (2020). Vision Loss in Optic Disc Drusen Correlates with Increased Macular Vessel Diameter and Flux and Reduced Peripapillary Vascular Density. Am. J. Ophthalmol..

[11] Xu B., Chen J., Zhang S., Shen S., Lan X., Chen Z., Yan Z., Xu B. (2021). Association between the Severity of Diabetic Retinopathy and Optical Coherence Tomography Angiography Metrics. Front. Endocrinol..

[12] Le D., Dadzie A., Son T., Lim J.I., Yao X. (2023). Comparative Analysis of OCT and OCT Angiography Characteristics in Early Diabetic Retinopathy. Retina.

[13] Xie Z., Zeinstra N., Kirby M.A., Le N.M., Murry C.E., Zheng Y., Wang R.K. (2024). Quantifying Microvascular Structure in Healthy and Infarcted Rat Hearts Using Optical Coherence Tomography Angiography. IEEE Trans. Med. Imaging.

[14] Agarwal A., Aggarwal K., Akella M., Agrawal R., Khandelwal N., Bansal R., Singh R., Gupta V., OCTA Study Group (2019). Fractal Dimension and Optical Coherence Tomography Angiography Features of the Central Macula after Repair of Rhegmatogenous Retinal Detachments. Retina.

[15] Yu S., Lakshminarayanan V. (2021). Fractal Dimension and Retinal Pathology: A Meta-Analysis. Appl. Sci..

[16] Engelmann J., Kearney S., McTrusty A., McKinlay G., Bernabeu M.O., Strang N. (2024). Retinal Fractal Dimension Is a Potential Biomarker for Systemic Health—Evidence From a Mixed-Age, Primary-Care Population. Transl. Vis. Sci. Technol..

[17] Ong S.S., Peavey J.J., Hiatt K.D., Whitlow C.T., Sappington R.M., Thompson A.C., Lockhart S.N., Chen H., Craft S., Rapp S.R. (2024). Association of Fractal Dimension and Other Retinal Vascular Network Parameters with Cognitive Performance and Neuroimaging Biomarkers: The Multi-Ethnic Study of Atherosclerosis (MESA). Alzheimer’s Dement..

[18] Kwon J., Choi J., Shin J.W., Lee J., Kook M.S. (2018). An Optical Coherence Tomography Angiography Study of the Relationship between Foveal Avascular Zone Size and Retinal Vessel Density. Investig. Ophthalmol. Vis. Sci..

[19] Ragkousis A., Kozobolis V., Kabanarou S., Bontzos G., Mangouritsas G., Heliopoulos I., Chatziralli I. (2020). Vessel Density around Foveal Avascular Zone as a Potential Imaging Biomarker for Detecting Preclinical Diabetic Retinopathy: An Optical Coherence Tomography Angiography Study. Semin. Ophthalmol..

[20] Vujosevic S., Cunha-Vaz J., Figueira J., Löwenstein A., Midena E., Parravano M., Peto T. (2021). Standardization of Optical Coherence Tomography Angiography Imaging Biomarkers in Diabetic Retinal Disease. Ophthalmic Res..

[21] Li Y.K., Fung N.S.K., Chan J.C., Choy B.N., Chow L.L., Shih K.C., Wong I.Y. (2023). OCTA Biomarkers in Adults Aged 50 and Above: A Prospective and Cross-Sectional Community-Based Study. BMC Ophthalmol..

[22] Hufendiek K., Lindziute M., Kaufeld J., Volkmann I., Brockmann D., Hosari S., Hufendiek K. (2023). Investigation of OCTA Biomarkers in Fabry Disease: A Long Term Follow-up of Macular Vessel Area Density and Foveal Avascular Zone Metrics. Ophthalmol. Ther..

[23] Kim K., Kim E.S., Yu S.Y. (2018). Optical Coherence Tomography Angiography Analysis of Foveal Microvascular Changes and Inner Retinal Layer Thinning in Patients with Diabetes. Br. J. Ophthalmol..

[24] Shiihara H., Terasaki H., Sonoda S., Kakiuchi N., Shinohara Y., Tomita M., Sakamoto T. (2018). Objective Evaluation of Size and Shape of Superficial Foveal Avascular Zone in Normal Subjects by Optical Coherence Tomography Angiography. Sci. Rep..

[25] Ersoz M.G., Hocaoglu M., Arf S., Karacorlu M. (2020). Macular Telangiectasia Type 2: Acircularity Index and Quantitative Assessment of Foveal Avascular Zone Using Optical Coherence Tomography Angiography. Retina.

[26] Piao H., Guo Y., Zhang H., Sung M.S., Park S.W. (2021). Acircularity and Circularity Indexes of the Foveal Avascular Zone in High Myopia. Sci. Rep..

[27] Werner J.U., Dreyhaupt J., Enders C. (2023). Evaluation of Automated Measurement of Macular Ischemic Changes in Retinal Vein Occlusion with Optical Coherence Tomography Angiography. Ophthalmic Surg. Lasers Imaging Retin..

[28] Mao J., Lin J., Zhu L., Liu C., Yu X., Zhang C., Chen Y., Zhang Y., Shen L. (2020). Quantitative Assessment of Retinal Capillary Vessel Density and Foveal Avascular Zone Area in Central Serous Chorioretinopathy Using OCTA. Ophthalmologica.

[29] DaCosta J., Bhatia D., Talks J. (2020). The Use of Optical Coherence Tomography Angiography and Optical Coherence Tomography to Predict Visual Acuity in Diabetic Retinopathy. Eye.

[30] Lemaître G., Rastgoo M., Massich J., Cheung C.Y., Wong T.Y., Lamoureux E., Milea D., Mériaudeau F., Sidibé D. (2016). Classification of SD-OCT Volumes Using Local Binary Patterns: Experimental Validation for DME Detection. J. Ophthalmol..

[31] Alfahaid A., Morris T., Cootes T., Keane P.A., Khalid H., Pontikos N., Sergouniotis P., Balaskas K. (2016). A Hybrid Machine Learning Approach Using LBP Descriptor and PCA for Age-Related Macular Degeneration Classification in OCTA Images. Annual Conference on Medical Image Understanding and Analysis.

[32] Li M., Chen Y., Ji Z., Xie K., Yuan S., Chen Q., Li S. (2020). Image Projection Network: 3D to 2D Image Segmentation in OCTA Images. IEEE Trans. Med. Imaging.

[33] Uji A., Murakami T., Nishijima K., Akagi T., Horii T., Arakawa N., Yoshimura N. (2012). Association between hyperreflective foci in the outer retina, status of photoreceptor layer, and visual acuity in diabetic macular edema. Am. J. Ophthalmol..

[34] Kaplan K., Kaya Y., Kuncan M., Ertunç H.M. (2020). Brain Tumor Classification Using Modified Local Binary Patterns (LBP) Feature Extraction Methods. Med. Hypotheses.

[35] Alam M., Zhang Y., Lim J.I., Chan R.V., Yang M., Yao X. (2020). Quantitative Optical Coherence Tomography Angiography Features for Objective Classification and Staging of Diabetic Retinopathy. Retina.

[36] Wang J., Rao C., Goh M., Xiao X. (2023). Risk assessment of coronary heart disease based on cloud-random forest. Artif. Intell. Rev..

[37] Chen T., Guestrin C. Xgboost: A Scalable Tree Boosting System. Proceedings of the 22nd ACM SIGKDD International Conference on Knowledge Discovery and Data Mining.

[38] Hancock J.T., Khoshgoftaar T.M. (2020). CatBoost for Big Data: An Interdisciplinary Review. J. Big Data.

[39] Ke G., Meng Q., Finley T., Wang T., Chen W., Ma W., Ye Q., Liu T.Y. Lightgbm: A Highly Efficient Gradient Boosting Decision Tree. Proceedings of the Advances in Neural Information Processing Systems.

[40] Cortes C., Vapnik V. (1995). Support-Vector Networks. Mach. Learn..

[41] Désir C., Petitjean C., Heutte L., Salaun M., Thiberville L. (2012). Classification of Endomicroscopic Images of the Lung Based on Random Subwindows and Extra-Trees. IEEE Trans. Biomed. Eng..

[42] Raj R., Mathew J., Kannath S.K., Rajan J. (2023). StrokeViT with AutoML for Brain Stroke Classification. Eng. Appl. Artif. Intell..

[43] Abiodun O.I., Jantan A., Omolara A.E., Dada K.V., Mohamed N.A., Arshad H. (2018). State-of-the-Art in Artificial Neural Network Applications: A Survey. Heliyon.

[44] Giannotti A., Lo Vecchio S., Musco S., Pollina L., Vallone F., Strauss I., Paggi V., Bernini F., Gabisonia K., Carlucci L. (2023). Decoding Bladder State from Pudendal Intraneural Signals in Pigs. APL Bioeng..

[45] Yasser I., Khalifa F., Abdeltawab H., Ghazal M., Sandhu H.S., El-Baz A. (2022). Automated Diagnosis of Optical Coherence Tomography Angiography (OCTA) Based on Machine Learning Techniques. Sensors.

[46] Cunha-Vaz J., Ribeiro L., Lobo C. (2014). Phenotypes and Biomarkers of Diabetic Retinopathy. Prog. Retin. Eye Res..

[47] Curtis T.M., Gardiner T.A., Stitt A.W. (2009). Microvascular Lesions of Diabetic Retinopathy: Clues towards Understanding Pathogenesis?. Eye.

[48] O’Leary F., Campbell M. (2023). The Blood–Retina Barrier in Health and Disease. FEBS J..

[49] Morgan J.I., Chui T.Y., Grieve K. (2023). Twenty-Five Years of Clinical Applications Using Adaptive Optics Ophthalmoscopy. Biomed. Opt. Express.

[50] Lin A., Fang D., Li C., Cheung C.Y., Chen H. (2020). Improved Automated Foveal Avascular Zone Measurement in Cirrus Optical Coherence Tomography Angiography Using the Level Sets Macro. Transl. Vis. Sci. Technol..

[51] Akıdan M., Erol M.K., Gedik B., Doğan M.E., Başol I., Süren E. (2024). Changes in Outcomes of Macular Optical Coherence Tomography Angiography Following Surgery for Optic Disc Pit Maculopathy. Diagnostics.

[52] Ahmadzadeh Amiri A., Sheikh Rezaee M.R., Ahmadzadeh Amiri A., Soleymanian T., Jafari R., Ahmadzadeh Amiri A. (2020). Macular Optical Coherence Tomography Angiography in Nephropathic Patients with Diabetic Retinopathy in Iran: A Prospective Case–Control Study. Ophthalmol. Ther..

